# Academic performance and social networks of adolescents in a caribbean city in Colombia

**DOI:** 10.1186/s40359-023-01299-9

**Published:** 2023-08-31

**Authors:** Milton López-Sánchez, Carlos Mario Arango-Paternina, Jose Petro-Petro, Lucía Lema-Gómez, Cleiber Eusse-López, Jorge Luis Petro, Willinton Watts-Fernández, Fabio Perea-Velásquez

**Affiliations:** 1 Research Group in Physical Activity, Sports and Health Sciences (GICAFS), Departamento de Cultura Física, Universidad de Córdoba, Avenue 6 #77- 305, Montería, Colombia; 2 Instituto Universitario de Educación Física, Universidad de Antioquia, Avenue 75 #65-87 - Bloque 45, Medellín, Colombia

**Keywords:** Education, Friendship, Social Network Analysis

## Abstract

**Background:**

Social factors and networks of friends can influence an adolescent’s behavior, including academic performance (AP) in school. This study aimed to analyze the relationship between AP and adolescents’ social networks in a Caribbean city in Colombia.

**Methods:**

A cross-sectional study was carried out with 806 schoolchildren from 12 to 17 years old of both sexes (52.7% girls), selected by multi-stage sampling from schools in the rural and urban areas of the city of Montería, Colombia. The AP was obtained from the school records; the sociodemographic variables included the location of the school (rural or urban), family structure, family functioning (Apgar score), and family affluence scale. Social network variables included social activity, popularity, reciprocity, homophily, friends’ academic performance, network size, network density, cluster of friends, and centrality.

**Results:**

The AP was inversely associated with the Apgar score in boys. No associations of AP with the school location, family structure, family affluence scale, and age were observed. In social network variables, AP was positively associated with popularity and friends’ academic performance in girls and boys, and negatively associated with homophily in boys.

**Conclusions:**

AP was associated with social network variables. These results could help implement interventions to improve adolescents’ social environment and AP.

## Background

Academic performance (AP) of students is an essential indicator of intellectual health, and it has been one of the leading indicators to evaluate the educational development of a country; therefore, improving AP levels is considered a fundamental part of the development of human capital in all nations [1]. Although AP is a concept that provokes debate (i.e., by its definition, factors that influence its development and evaluation), the AP expresses the progress or “performance” of students in a particular area or academic situation through a grade which is assigned based on an evaluation system [2, 3]. In this context, standardized academic tests employed to evaluate the AP level serve as an input to draw educational policies and strategies and compare the level of educational development between countries [4]. In this sense, there is concern about the educational level of Latin America because the AP (i.e., reading, mathematics, and science) is inferior compared to the AP average of countries of the Organisation for Economic Co-operation and Development (OECD) [5]. This panorama imposes the need to evaluate the factors associated with AP under the sociodemographic conditions of Latin American countries, which contributes to the management and development of strategies to improve the AP of students [6].

As a complex phenomenon, AP is influenced by multiple factors, such as personal, economic, educational, and social aspects [7–9]. Particularly, previous research has indicated that the social networks of friends may influence the behaviors of adolescents (e.g., alcohol consumption, tobacco, and physical activity) [10, 11], including the AP [12–16]. Frequent social interaction within groups enables influence through encouragement, advice, and group pressure. Social interaction is also an important element for adolescents because, within these networks, they find affection, information, and recognition. Simultaneously, they create new social connections, shape subcultures and behavioral norms [17]. Children and adolescents spend much of their lives interacting with their peers at school. In these interactions, social networks are forged, norms are structured, and behaviors are influenced [18]. Particularly during adolescence, when family ties gradually become less influential [19] and the network of friends is expanded [20]. These friendly relationships with peers are also related to academic performance [16], relationships that can be positive or negative [21]. Previous research has indicated that belonging to a group of friends can positively serve academic success; schoolchildren who perceive themselves as socially isolated have lower academic performance than those who feel supported by their friends [22]. In this sense, adolescents who enjoy positive relationships with their peers tend to excel academically in response to various mechanisms of social influence [23]. Therefore, school social structures are valuable for school performance and adaptation. The social relationships established and sustained within the school represent one of the most important facets of the schooling process.

Social network analysis is a sound theoretical and analytical tool to investigate the structure of the group of friends with whom the individual interacts in socialization environments [24], such as at school. This tool helps to analyze the network actors’ connection patterns, allowing the identification of different sociometric attributes of the structure of the social network of friends [25]. Social network analysis also enables the use of network data to predict the strength of ties between individuals through those attributes and the level of influence exerted on individual behaviors [26]. For example, social network analysis enables identifying those actors who tend to receive a more significant number of friendship nominations within the network (popular actors) [25], and those who tend to make more friendship nominations, an indicator of high social activity. As well, the degree of similarity between the individuals that are interconnected in the network, which is known as homophily [27]. It is also possible to identify network size and density, clusters of friends, and actors with a centralized or peripheral position within the network, among other sociometric parameters [25].

Knowing the structure of the social network is critical to understanding the dynamics of collective entities, such as social support, social influence, or social capital, entities of particular interest for school performance [28]. By applying social network analysis to explore a complex phenomenon such as AP in adolescence, the understanding of AP is broadened from a multidisciplinary perspective, which could contribute to formulating educational policies and strategies. However, in Latin American countries, such as Colombia, which face critical educational quality challenges [29], studies of social networks in aspects related to school performance are scarce. Considering these aspects, we hypothesize that network measures will be associated with AP. Therefore, the purpose of this study was to analyze the association between AP with social networks in adolescents from a Caribbean city in Colombia.

## Methodology

### Design and sample

This study is part of the research project “Determinants of academic achievement, health, and wellness in school-age children” (De-Redes). The study was conducted with a cross-sectional design in adolescents aged 12 to 17 years from a Caribbean city in Colombia. The study complied with the guidelines stipulated in the Declaration of Helsinki, and its protocol was approved by an ethics committee of Universidad de Antioquia (No. 2017-021). Multi-stage sampling was carried out in a sample frame of around 80,000 schoolchildren registered in 63 public schools in the city for 2018 (80% in the urban sector), of which 30% were schoolchildren in grades 7 and 10. Ten schools were randomly selected, and two did not allow access to the students’ academic performance records. In the schools included, one classroom was randomly selected in each grade (7th through 10th), resulting in 32 classrooms in the eight schools. Students from these classrooms were invited to participate in the study (n = 1113) and received informed assent, and their parents received informed consent. Students who returned the signed informed assent and consent forms were included in the study. Students with missing data were excluded from the analysis. The total study sample consisted of 806 adolescents.

### Measurements

#### Academic performance

The academic performance variable was obtained by consulting school records. In them, a scale from 1 to 10 was used to assess academic performance, and for this study, the academic grade point averages were obtained in all school courses.

*Sociodemographic variables*: The school’s location (urban or rural), the sex, and the age of each student were recorded. For the family aspects, the students reported the type of family structure taking into account three categories: nuclear family, made up of both parents; single-parent family, for those students who reported living with one of the parents; and another family structure, for those families other than nuclear and single-parent families. Additionally, participants filled out the validated Apgar instrument, which has good internal consistency with Cronbach’s alpha coefficient of 0.81 and McDonald’s omega coefficient of 0.82 [30]. The student’s perception of the functioning of the family in five dimensions was assessed (one item for each one): adaptation, cooperation, growth, affectivity, and problem-solving. Each dimension had a score from 0 to 5, and the final score ranged from 0 to 25. The Family Affluence Scale (FAS) was used to obtain a proxy of economic position [31]. FAS has good criterion validity reported in previous studies (Spearman correlation = 0.87 and kappa index = 0.57) [32]. This scale is composed of four questions inquiring about the number of cars and computers in the home, internet accessibility at home, and whether the student has his own bedroom, with a scale of none = 0; one = 1; two or more = 2. The total score ranged from 0 to 8.

#### Social network variables

To identify the social network of friends, the students received a list with the names of their classmates to nominate their best friends within the classroom, with no limit on the number of nominations. The participants were instructed that best friends were those with whom they have a close friendship, share activities frequently, and have common interests in some activities. This specification was based on the recommendation made in the friendship literature [33] to distinguish between best friends, friends, and peers. With these nominations, an egocentric analysis of the social network of friends was carried out, calculating the following social network variables, considered of utility in this type of network analysis [34].

1. *Social activity* reflects the social expansiveness of the student and is extracted by counting the number of nominations made.
2. *Popularity* indicates the level of reputation of the student within the social network of friends and is extracted by counting the number of nominations received.
3. *Reciprocity* reflects the level of closeness of friendship ties and is based on the percentage of reciprocal nominations; that is, when A nominates B and B nominates A.
4. *Homophily* reflects the student’s tendency to nominate friends with whom the level of academic performance is shared (similarity) and is defined as the average of the absolute differences in the individual AP and the AP of their friends.
5. *Average academic performance of friends* indicated the magnitude of academic performance surrounding the student and was calculated by averaging the academic performance of the student’s friends.
6. *Network size* reflected the breadth of the social network of friends and was calculated by counting the number of classmates with whom the student has friendship ties.
7. *Network density* indicated the degree of connectivity within the personal network of friends and was calculated by dividing the network size by the number of potential connections.
8. *Cluster of friends* reflected the student’s tendency to form friendship triangles and were defined by the number of times the student was part of triads of friends.
9. *Centrality* indicated the level of importance of the actor within the network in terms of connectivity. It was calculated by counting the number of times the student was on the shortest route between two members of the network of friends.

Since all these network variables had a non-normal distribution, they were transformed using the two-step approach [35].

### Analysis plan

Data normality was tested by the Kolmoronov-Smirnov test. Descriptive statistics were stratified by sex. Proportions, means, and standard deviation were analyzed using the chi-square test for categorical variables and the t-test for continuous variables. Associations between social network variables and academic performance were analyzed with bivariate and multivariate linear regression models stratified by gender. Confidence intervals (95%) were estimated; the level of significance was established at p-value < 0.05, and the effect size was calculated based on Cohen’s *f*^*2*^ [36], both in bivariate and multivariate associations. The Homer-Lemeshow criterion [37] was applied for the multivariate models so that the variables with p-values < 0.25 in the bivariate associations were included in the multivariate linear regression models. Social network variables were obtained using the UCINET program [37]. The SPSS Statistical Program for Windows v.24 (IBM Corp., Armonk, N.Y., USA) was used to conduct the analyses.

## Results

In this study, the sample consisted of 806 adolescents (12 to 17 years old) of both sexes (n = 425, 52.7% girls), belonging to schools in urban and rural areas (n = 637, 79% urban) of the city ​​of Monteria, Colombia. Most of these students reported that they belonged to a nuclear-type family structure (n = 538, 66.75%), with no differences between boys and girls in the distribution of the different types of family structure. The score on the family affluence scale and family functioning (Apgar score) showed no difference according to sex (*p* = 0.166 and *p* = 0.125, respectively). However, girls had higher AP than boys (*p* = 0.001). Regarding the variables of the social network, differences were found between boys and girls in reciprocity (*p* = 0.008), homophily (*p* = 0.001), the academic performance of friends (*p* = 0.005), and network density (*p* = 0.003). No differences were found between boys and girls in the other network variables. These results are presented in Table 1.

**Table 1:** Sample characteristics

Variables	Girls, n = 425 (52.7%)	Boys, n = 381 (47.3%)	*p*-value
Location	n (%)	n (%)	
Urban	338 (79.5)	299 (78.5)	0.714
Rural	87 (20.5)	82 (21.5)	
Family structure, n (%)			
Other	40 (9.4)	24 (6.3)	0.264
Single parent	106 (24.9)	98 (25.7)	
Nuclear	279 (65.6)	259 (68.0)	
	Mean (SD)	Mean (SD)	
Age	14.8 (1.3)	15 (1.4)	0.027
Family Affluence Scale	1.8 (1.5)	1.9 (1.7)	0.166
Apgar score	13.9 (4.5)	14.3 (3.7)	0.125
Academic performance	7.3 (0.7)	7.1 (0.7)	0.001
Network variables			
Social activity	8.9 (6.3)	8.9 (6.3)	0.965
Popularity	8.8 (4.6)	9 (4.5)	0.734
Reciprocity	0.42 (0.21)	0.38 (0.2)	0.008
Homophily	-0.03 (0.05)	-0.05 (0.06)	0.001
Academic performance of friends	7.3 (0.4)	7.3 (0.4)	0.005
Network size	12.4 (6.3)	12.7 (5.8)	0.480
Network density	50.8 (17.6)	47.3 (16.2)	0.003
Cluster of friends	91.7 (92.6)	91.6 (89.2)	0.996
Centrality	15.4 (21.0)	18.2 (20.3)	0.053

The bivariate association analysis between AP and sociodemographic variables showed that there was an association between AP and the school location (i.e., rural area as reference), with a small effect size, in adolescents of both sexes (girls: *p* < 0.01, *f*^*2*^ = 0.12, boys: *p* < 0.01, *f*^*2*^ = 0.08). Considering family structure (i.e., ‘other’ as a reference), no significant association was found between AP and single-parent or nuclear structure in girls or boys. In contrast, age was inversely associated with AP with a small effect size in both sexes (girls: *p* < 0.01, *f*^*2*^ = 0.03; boys: *p* < 0.01, *f*^*2*^ = 0.05). On the other hand, the Family Affluence Scale and the Apgar score were not associated with AP in girls (*p* = 0.09, *f*^*2*^ = 0.01 and *p* = 0.31, *f*^*2*^ = 0.00, respectively). However, this association did occur in boys with a small effect size (*p* = 0.046, *f*^*2*^ = 0.01 and *p* = 0.001, *f*^*2*^ = 0.03). Regarding the social network variables, the association between AP and popularity was positive, with a small effect size in girls (*p* < 0.01, *f*^*2*^ = 0.03). A positive association was found between AP and the academic performance of friends, presenting a large effect size in girls (*p* < 0.01, *f*^*2*^ = 0.38) and boys (*p* < 0.01, *f*^*2*^ = 0.40). Similarly, it occurred with network size (*p* = 0.01, *f*^*2*^ = 0.01, for girls, and *p* = 0.019, *f*^*2*^ = 0.01, for boys), cluster of friends (*p* = 0.04, *f*^*2*^ = 0.01, for girls) and centrality (*p* = 0.02, *f*^*2*^ = 0.01 for girls, and *p* < 0.01, *f*^*2*^ = 0.06, for boys). Network variables such as homophily and network density were not associated with PA in girls (*p* = 0.83, *f*^*2*^ = 0.00 and *p* = 0.34, *f*^*2*^ = 0.00, respectively), but in boys, showing a small effect size (*p* = 0.002, ƒ^*2*^ = 0.02 and *p* < 0.01, *f*^*2*^ = 0.04, respectively). For their part, social activity and reciprocity were the only network variables that did not present an association with AP in girls or boys (Table 2).

**Table 2:** Bivariate associations of academic performance with sociodemographic and network variables

Variables	Girls			Boys		
	β (95% CI)	*p-value*	*Cohen’s f* ^2^	β (95% CI)	*p-value*	*Cohen’s f* ^2^
School location (Ref. Rural)	0.56 (0.40–0.71)*	0.00	0.12	0.45 (0.29–0.61)*	0.00	0.08
Family structure (Ref. Other)						
Single parent	-0.05 (-0.29–0.20)	0.72	0.00	-0.13 (-0.43–0.17)	0.39	0.00
Nuclear	-0.01 (-0.24–0.22)	0.93		-0.11 (-0.39–0.17)	0.44	
Age	-0.08 (-0.13 - -0.04)*	0.00	0.03	-0.10 (-0.15 - -0.06)*	0.00	0.05
Family Affluence Scale	0.04 (-0.01–0.08)	0.09	0.01	0.04 (0.001–0.08)*	0.046	0.01
Apgar score	0.01 (-0.01–0.02)	0.31	0.00	0.03 (0.01–0.05)*	0.001	0.03
Network variables						
Social activity	0.01 (-0.001–0.02)	0.08	0.01	0.01 (-0.002–0.02)	0.108	0.01
Popularity	0.03 (0.01–0.04)*	0.00	0.03	0.01 (-0.001–0.03)	0.060	0.01
Reciprocity	0.19 (-0.11–0.49)	0.21	0.00	0.20 (-0.15–0.54)	0.260	0.00
Homophily	-0.15 (-1.44–1.15)	0.83	0.00	-1.70 (-2.79 - -0.61)*	0.002	0.02
Academic performance of friends	0.84 (0.71–0.97)*	0.00	0.38	0.91 (0.77–1.06)*	0.000	0.40
Network size	0.01 (0.002–0.02)*	0.02	0.01	0.01 (0.002–0.03)*	0.019	0.01
Network density	-0.002 (-0.01–0.002)	0.34	0.00	-0.01 (-0.01 - -0.004)*	0.000	0.04
Cluster of friends	0.001 (0.001–0.01)*	0.04	0.01	0.003 (-0.00–0.001)	0.464	0.00
Centrality	0.004 (0.001–0.007)*	0.02	0.01	0.008 (0.005–0.011)*	0.000	0.06

* Statistically significant association (p < 0.05)

In the analysis of the multivariate models of association with AP, the variables that obtained a p-value < 0.25 in the bivariate associations were included, according to the Homer-Lemeshow criterion [38]. Thus, the analysis of these models showed a large effect size in adolescents of both sexes (ƒ^*2*^ = 0.49 in girls and ƒ^*2*^ = 0.57 in boys). Of the sociodemographic variables included, only a positive association was found between AP and Apgar score in boys (*p* < 0.01). In the case of the network variables, a significant association of AP was found with popularity (girls: *p* < 0.01; boys: *p* = 0.02), the academic performance of friends (p < 0.01 in both sexes), and cluster of friends (*p* < 0.1 in both sexes); while the variable homophily was negatively associated with AP in boys (*p* = 0.01) in this model (Table 3).

**Table 3:** Multivariate associations of academic performance with sociodemographic and network variables

Variables	Girls			Boys		
	β (95% CI)	*p-value*	*Cohen’s f* ^2^	β (95% CI)	*p-value*	*Cohen’s f* ^2^
School location (Ref. Rural)	0.11 (-0.05–0.27)	0.19	0.49	0.01 (-0.15–0.17)	0.90	0.57
Family structure (Ref. Other)						
Single parent	----	----		----	----	
Nuclear	----	----		----	----	
Age	-0.03 (-0.07–0.011)	0.14		-0.04 (-0.08–0.01)	0.09	
Family Affluence Scale	-0.01 (-0.05–0.02)	0.48		0.01 (-0.03–0.04)	0.65	
Apgar score	----	----		0.02 (0.01–0.04)*	0.00	
Network variables						
Social activity	0.03 (-0.01–0.06)	0.13		0.01 (-0.01–0.03)	0.43	
Popularity	0.06 (0.02–0.11)*	0.00		0.04 (0.01–0.07)*	0.02	
Reciprocity	-0.03 (-0.47–0.42)	0.90		----	----	
Homophily	----	----		-1.27 (-2.21 - -0.34)*	0.01	
Academic performance of friends	0.77 (0.62–0.92)*	0.00		0.79 (0.63–0.95)*	0.00	
Network size	0.01 (-0.06–0.08)	0.78		-0.03 (-0.06–0.01)	0.11	
Network density	----	----		-0.01 (-0.01–0.0003)	0.07	
Cluster of friends	-0.004 (-0.008–0.0)	0.10		----	----	
Centrality	-0.003 (-0.009–0.0)	0.33		0.003 (-0.003–0.008)	0.32	

* Statistically significant association (p < 0.05)

## Discussion

The objective of this study was to analyze the relationship between academic performance (AP) and social network variables in adolescents. The results of the multivariate models indicated that some social network variables were significantly associated with AP; that is, links between social dynamics and adolescent school performance were demonstrated. For example, the study documents that popular students (those with higher friendship nominations received) had higher AP. This finding suggests that adequate AP is a socially valued attribute in school networks. In other words, acceptable AP may give the adolescent a certain status and visibility within these networks of friends. Although in previous studies, popularity had also been associated with negative attributes such as risk behaviors [39] and aggressive behaviors [40]. This indicated that popularity is a complex construct and depends on the social context in which it is manifested.

Another study finding was that friends’ academic performance was reflected in adolescents’ academic performance. This would indicate that the academic environment in the group of friends, whether adequate or deficient, seemed to influence adolescent performance in the same way as previously documented [41]. A group of friends is an important source of social support, and seeking help in the classroom promotes learning and social skills, leading to better academic performance in a group of friends. However, this process is influenced by the same academic and social environment [42] and by variables such as gender. In this regard, it has been pointed out that academic performance tends to be more similar between same-sex and opposite-sex pairs [43]. This finding indicates that the social dynamics among the groups of friends represent the possibility of accessing necessary resources for school performance [44].

Another network parameter analyzed in the study was homophily, understood as the tendency to establish social bonds with similar people, an elementary principle of interpersonal relationships [27]. The study found that, in boys, students with higher AP tended to have more friends with similar AP (homophily). Previous studies have reported that students tend to nominate and be nominated by peers with similar academic performance [14, 45]. Likewise, it has been documented that homophily is present in schoolchildren with low school attendance and, consequently, with low academic performance [46]. Homophily can arise due to selection, socialization processes, or influence [47]. In this regard, a longitudinal study found that homophily in academic performance results from a selection process and not from influence [48]. Finally, although this study did not find an association between network size and reciprocity with academic performance, previous studies have documented these associations [49–51].

These findings highlight the complexity of social dynamics and interactions within school groups, and these results have implications for practice. The social agenda accompanying students in educational meetings could be used positively to enhance the achievement of learning objectives, favoring teaching models that emphasize social interaction, such as cooperative learning, group learning, or teamwork. The implications for research have to do with the need to reveal the effects of social network interventions on the academic performance of adolescents. Also, future studies may include other agents of socialization outside the school to have a complementary component of the social circle of adolescents.

The study has some weaknesses. First, since this is a cross-sectional design study, any inference of causality between network characteristics and academic performance is ruled out. Second, networks of school friends were analyzed in the study. Other socialization settings outside of school were not studied. Third, although academic performance was taken as the outcome variable, the association may be in the opposite direction; that is, academic performance is the factor that promotes the establishment of social bonds among friends. And fourth, we have analyzed the relationship between social network parameters and academic performance by applying egocentric network analysis. Perhaps a socio-centric approach could have yielded results showing the mutual influence between AP and network ties.

## Conclusions

The study concludes that the variables popularity, the academic performance of friends, and cluster of friends were positively associated with academic performance in girls and boys, and that homophily and network size were negatively associated with AP in boys. These findings highlight the need to consider the configuration of social networks of friends within the classroom and its relationship with academic performance. Intervention studies are also required to reveal the effects of social networks on academic performance.

## Data Availability

The datasets generated and analyzed during the current study are available from the corresponding author upon reasonable request.
